# Video Feedback and Video Modeling in Teaching Laparoscopic Surgery: A Visionary Concept from Kiel

**DOI:** 10.3390/jcm10010163

**Published:** 2021-01-05

**Authors:** Ibrahim Alkatout, Juhi Dhanawat, Johannes Ackermann, Damaris Freytag, Göntje Peters, Nicolai Maass, Liselotte Mettler, Julian Maria Pape

**Affiliations:** Department of Gynecology and Obstetrics, University Hospitals Schleswig-Holstein, Campus Kiel, Arnold-Heller Str. 3, Building 24, 24105 Kiel, Germany; juhidhanawat@gmail.com (J.D.); johannes.ackermann@uksh.de (J.A.); Damaris.Freytag@uksh.de (D.F.); goentje.peters@uksh.de (G.P.); nicolai.maass@uksh.de (N.M.); profmettler@gmx.de (L.M.); JulianMaria.Pape@uksh.de (J.M.P.)

**Keywords:** video feedback, video modeling, laparoscopy, gynecology, surgical training, pelvitrainer

## Abstract

Learning curves for endoscopic surgery are long and flat. Various techniques and methods are now available for surgical endoscopic training, such as pelvitrainers, virtual trainers, and body donor surgery. Video modeling and video feedback are commonly used in professional training. We report, for the first time, the application of video modeling and video feedback for endoscopic training in gynecology. The purpose is to present an innovative method of training. Attendees (residents and specialists) of minimally invasive surgery courses were asked to perform specific tasks, which were video recorded in a multimodular concept. Feedback was given later by an expert at a joint meeting. The attendees were asked to fill a questionnaire in order to assess video feedback given by the expert. The advantages of video feedback and video modeling for the development of surgical skills were given a high rating (median 84%, interquartile ranges (IQR) 72.5–97.5%, *n* = 37). The question as to whether the attendees would recommend such training was also answered very positively (median 100%, IQR 89.5–100%, *n* = 37). We noted a clear difference between subjective perception and objective feedback (58%, IQR 40.5–76%, *n* = 37). Video feedback and video modeling are easy to implement in surgical training setups, and help trainees at all levels of education.

## 1. Introduction

Endoscopic surgery is available in all developed countries and is widely accepted in all surgical specialties. Its rapid growth and acceptance is seen even in developing countries. The acquisition of skills needed for endoscopic surgery involves a long learning curve. Training programs for laparoscopic surgery are required to fulfil the challenge of imparting a variety of surgical skills [1,2]. Laparoscopic surgery calls for refined psychomotor skills, which differ from those required for conventional surgery [3]. The challenges of laparoscopic surgery include the fact that a two-dimensional image is extrapolated to a three-dimensional working area, the fulcrum effect, specialized coordination of hands and eyes, depth perception, and a different type of tactile feedback. These skills can only be acquired and developed outside the operating room [4]. Thus, the surgeon completes the learning curve in an outsourced setting [5,6].

The traditional concept of teaching open surgery in the operating room [7] has now shifted to training schools for teaching and learning minimal access surgery [8,9]. It leads to superior improvement in knowledge and technical performance in the operating room, compared with conventional residency training [10].

A variety of options are available for acquiring skills in laparoscopic surgery, such as watching video films of operations, surgery on body donors and animals, practicing on laparoscopic trainers (pelvitrainers), and virtual reality simulators. Virtual trainers are the only devices that provide direct feedback and an evaluation of the surgeon’s exercises in laparoscopic training. Minimally invasive surgery requires the interaction of various components. Critical factors, in addition to the previously mentioned and known challenges, include weight distribution, body position, handling, and skilled static and pivotal movements. This, in turn, necessitates modular training on an individual basis.

Feedback is a crucial aspect of any method of training. The aim of feedback is to achieve a positive and constructive improvement in the acquisition of a particular skill. However, feedback about simple observations and achievements are very subjective and unable to meet the demands of fairness and objectivity [11]. A meta-analysis of 33 published empirical studies confirmed the effectiveness of video-supported feedback on the interaction skills of professionals in a variety of professions [12]. According to Hazen et al., simply pointing out mistakes is not enough to improve the skills of athletes [13]. Watching oneself highlights the positive aspects of one’s performance in addition to enhancing the learner’s motivation and the success of learning [14]. Learners are able to view their pre-existing repertoire of skills. An additional advantage of observational or model learning is that complex modes of behavior, including a large number of behavior patterns, can be acquired more easily and rapidly [15]. Video enhancement was used as an evidence based and reliable tool for the in-training assessment of residents non-technical performance in the operation room [16].

A skill that appears easy at first glance is difficult when performed. However, training the mind is propagated as a means of achieving anything the mind desires. Psychological training of the mind is also important for the refinement of motor functions.

Training on pelvitrainers with subsequent feedback based on video recordings, in conjunction with a comparison of an expert’s skills, was shown to be a promising additional means of learning. In 1963, video feedback was used for the first time in communication training [17]. Watt reported a significant improvement of speech as a result of video feedback [18]. Kurtz et al. described video feedback as a gold standard in communication training [19]. The method spread rapidly and successfully to other areas such as first-aid training, sports medicine for primary injury prophylaxis, and parent training programs [20,21,22,23]. The benefits of video feedback are very evident in sports. It has been used for the improvement of skills in martial arts, basketball, soccer, swimming, and tennis [24,25] [26]. The addition of a film showing an expert performing the skill is the principle of video modeling [27]. Video feedback and video modeling show the participants their mistakes as well as their correct execution of the skill. Technical options permit visual recording of a task, freezing a particular image in time, and replaying it several times. Like sports, laparoscopic surgery requires a high level of psychomotor skills.

Based on the concept of using video feedback in endoscopic training, in the present pilot study we analyze the feasibility of video feedback and video modeling in a preexisting endoscopic training setup. The impact of such training on the trainees’ skills, and the target group that benefits most from additional video feedback and video modeling are also addressed.

## 2. Experimental Section

### 2.1. Participants and Training Setup

We collected data from persons who attended a minimally invasive surgery training program for beginners and advanced surgeons at the Kiel School of Gynecological Endoscopy (department of obstetrics and gynecology, University Hospital Schleswig-Holstein, Campus Kiel, Germany) between November 2019 and July 2020. The courses were certified by the German Society of Gynecological Endoscopy (AGE). None of the participants were students, residents, or consultants at the University Hospital Schleswig-Holstein, Campus Kiel. The participants were told that their tasks would be filmed for evaluation of their performance, and appropriate feedback would be given by experts. All participants consented to the procedure.

The attendees trained on a Realsimulator 2.0 (Endodevelop, Saarbrücken, Saarland, Germany) based on the female physique from the Pelvic School of Saarbrücken, and a SZABO-BERCI-SACKIER pelvitrainer from Karl Storz Company (KARL STORZ GmbH and Co. KG in Tuttlingen, Baden-Württemberg, Germany). The endoscopy system was provided by Karl Storz Company. The attendees used instruments from Karl Storz Company, consisting of the Clickline series (Manhes, Metzenbaum scissors) and two Koh needle holders.

**Figure 1:**
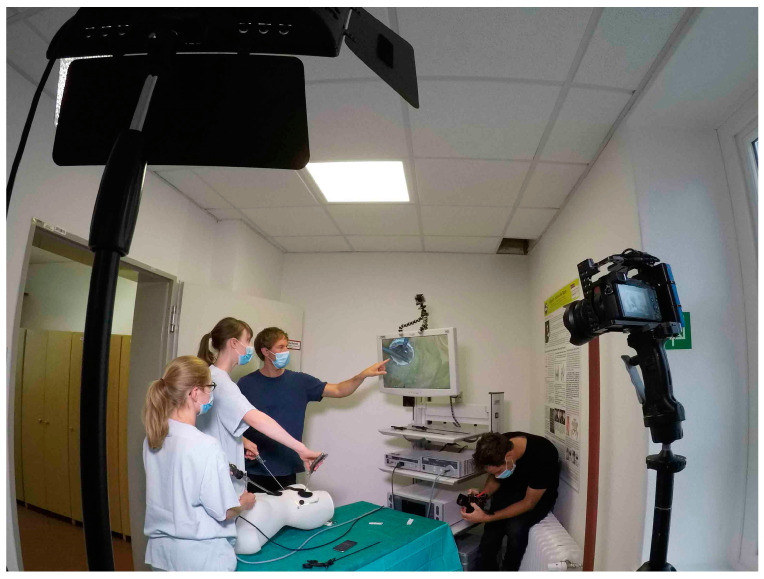
Setup of video feedback.

The workplace was equipped with a camera (Panasonic LUMIX Gh5 with a fixed focal length lens Panasonic Summilux 1:1.4/25 (Panasonic, Osaka, Japan)) installed on a tripod, which captured the handling and posture of the surgeon as well as the camera assistant (Figure 1, Video S1).

**Figure 2:**
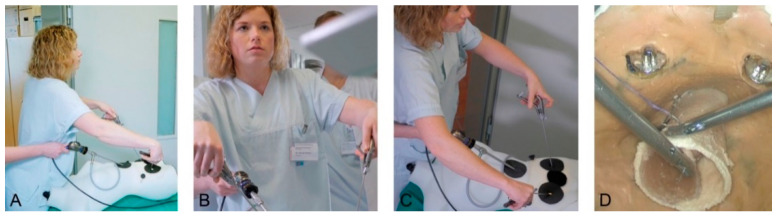
Four camera perspectives: posture (**A**), posture and instruments (**B**,**C**), and the surgical site (**D**).

The use of instruments was recorded precisely by a camera (Panasonic LUMIX G81 with Panasonic G Vario 1:3.5-5.6/12-60), which filmed alternately from above and in front of the participants (Figure 2). Additional lighting permitted the acquisition of high-quality video recordings. The endoscopic camera recorded the task being performed. All three cameras yielded high definition images. The three video recordings were synchronized and configured as a split screen to compare posture and instrument use with intracorporeal work, and compare it with the expert’s recordings. A filmmaker experienced in the use of video feedback in professional sports (such as surfing) accompanied the three- to four-day training courses, provided the necessary equipment, and created a personalized video clip for each participant.

The recordings were collected on a data storage device and processed with a video editing program (Adobe Premiere Pro cc, (Adobe, San Jose, CA, USA)). Table 1 provides a precise list of the equipment, video cutting and special software, and their costs.

### 2.2. Task Performance

The participants trained in twos on the pelvitrainer—alternately, as a surgeon and as a camera assistant. At the beginning of the endoscopic work, all participants were instructed in the execution of the exercises and the use of instruments. A task list, an instructional video performed by one of the experts, and oral instruction were given to each one. Any questions or ambiguities during the work were clarified by an expert. Suggestions for improvement and additions were offered. The time taken for each exercise was measured until the exercise was evidently completed. Two tasks were performed during the video feedback. Based on previous studies [28], we selected tasks from all surgical fields with respect to hand-eye coordination, posture, ergonomics, instrument handling, depth perception, and precision.

On the first day of the training program, each attendee of the course performed a resection task. A mark was made on a latex glove. The glove was fixed to a cork board with tacks. This, in turn, was fixed to the floor of the SZABO-BERCI-SACKIER pelvitrainer with Velcro. The marked figure had to be excised precisely. Cutting was only permitted in the upper layer of the glove. The time taken to complete the task was measured.

On the second day, all attendees made a suture and performed an intracorporeal knot on the vaginal vault, which is regarded as a complex task in laparoscopic surgery. An artificial vagina was fixed in an artificial pelvic model of the pelvic trainer aligned to the female physique. Two needle holders and a circular needle with a thread shortened to 15 cm were used. The time taken by each participant to perform one surgical knot was measured.

### 2.3. Video Feedback and Video Modeling

**Figure 3:**
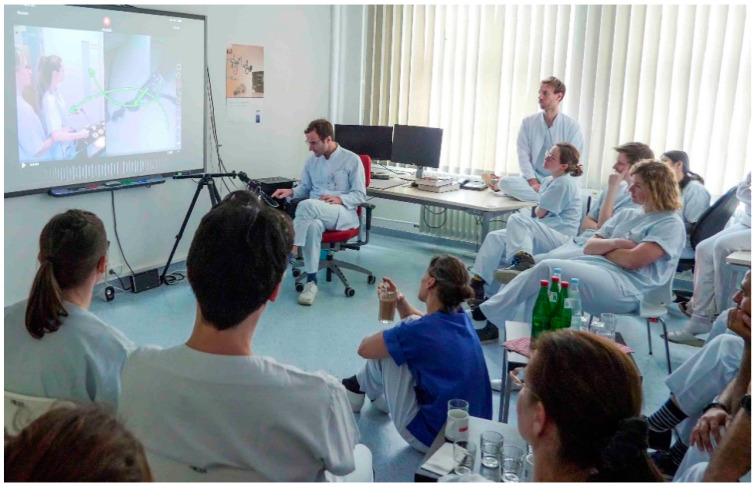
Setting of the video feedback assessment.

**Figure 4:**
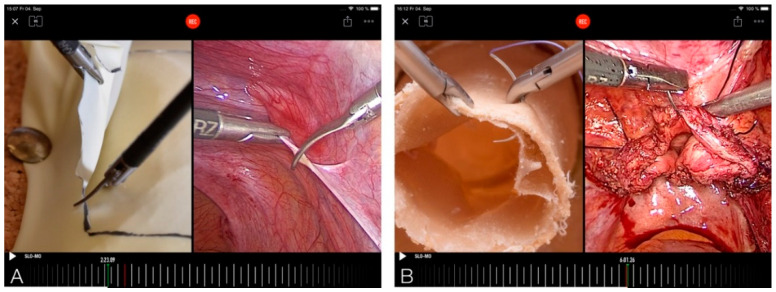
Video modeling of the resection task (**A**); video modeling of the vaginal vault closure task (**B**).

The video feedback assessment was given individually to each participant with all participants present at one place. Thus, each participant could also learn from the evaluation of other participants by two experienced endoscopic surgeons (Figure 3, Video S1: Video feedback and video modeling clip). The experts were two senior clinicians (I.A. and G.P.) with more than 15 years of experience in the field of minimally invasive surgery. They both perform approximately 800 surgeries per year. Each attendee received a 7- to 10-min video feedback on the two exercises by both the experts together. Recordings from the operating room, such as resection of a part of the peritoneum or closure of the vaginal vault, were used to compare the skills of the participants performing the above mentioned exercises (Figure 4A,B). The software named Coach’s Eye permits the user to play the video frame by frame in slow motion. In addition to verbal feedback, the experts could make visual corrections by manual input on the iPad. This was useful to point out improper posture or unsuitable handling of the instruments, and their improvement through visualization. The video, including vocal and visual feedback, was displayed on a screen and recorded. The feedback given by the experts was oriented to the OSATS score (Objective Structured Assessment of Technical Skills) [29]. At the end of the feedback, all attendees were asked to complete a modified self-formulated questionnaire based on questions already used in a previous study done by our group and approved by a statistician [30].

The attendees’ basic assessment of the course, the practical success of learning, and sociodemographic data were recorded in a standardized questionnaire. The attendees were asked to provide their self-assessment after the video feedback. They were also asked whether the course had been of any benefit, whether they would recommend it as a new training concept, and how it relates to the subjective improvement of posture and the overall task. The attendees had to answer the questions on a visual analog scale (VAS) of 10 cm. The answers were expressed in percentages (0—not useful to 100—very useful).

### 2.4. Statistics

All answers to the items in the questionnaire were tabulated in a Microsoft© Excel database (Microsoft Corp., Redmond, WA, USA). The IBM SPSS Statistics 23 program (IBM, Armonk, NY, USA) was used for statistical analysis. Quantitative variables were presented descriptively as means and standard deviations, minimum, maximum, quartiles and, interquartile ranges (IQR), and tested for normality with the Kolmogorov–Smirnov test. VAS scores were evaluated as follows: <20—very low; 20 to <40—low; 40 to <60—moderate; 60 to <80—high; and 80 to 100—very high. A correlation analysis was performed to determine the influence of age and the number of live surgery events attended in the past. When significant deviations from normal distribution were found, we used Spearman’s rho test for the correlation analysis. The correlation coefficient (r) was evaluated as follows: r ≤ 0.2—no correlation; 0.2 < r ≤ 0.5—weak to moderate correlation; 0.5 < r ≤ 0.8—strong correlation; and 0.8 < r ≤ 1.0—very strong correlation. Tests were performed bilaterally and the level of significance was set to 5% (*p* < 0.05). The Mann–Whitney U test was used for subgroup analysis of nonparametric data, or the Kruskal–Wallis test for more than two subgroups. Tests were performed bilaterally and the level of significance was set to 5% (*p* < 0.05).

## 3. Results

Thirty-seven persons participated in the study, of which 26 were female and 11 male. Twenty-six were resident doctors and 11 were specialists. Twenty-six persons had attended a minimally invasive surgery course in the past. Table 2 shows descriptive statistics regarding age (median: 33 years and range: 25–56 years) and the number of years of professional experience (median: 4 years and range: 0–40 years). The attendees had a median of 3.8 years of work experience, but the duration of their experience in minimally invasive surgery was only two years. In the self-assessment, the median value for experience as a surgeon in minimally invasive surgery was 10%, and the interquartile range was 3–40%.

**Figure 5:**
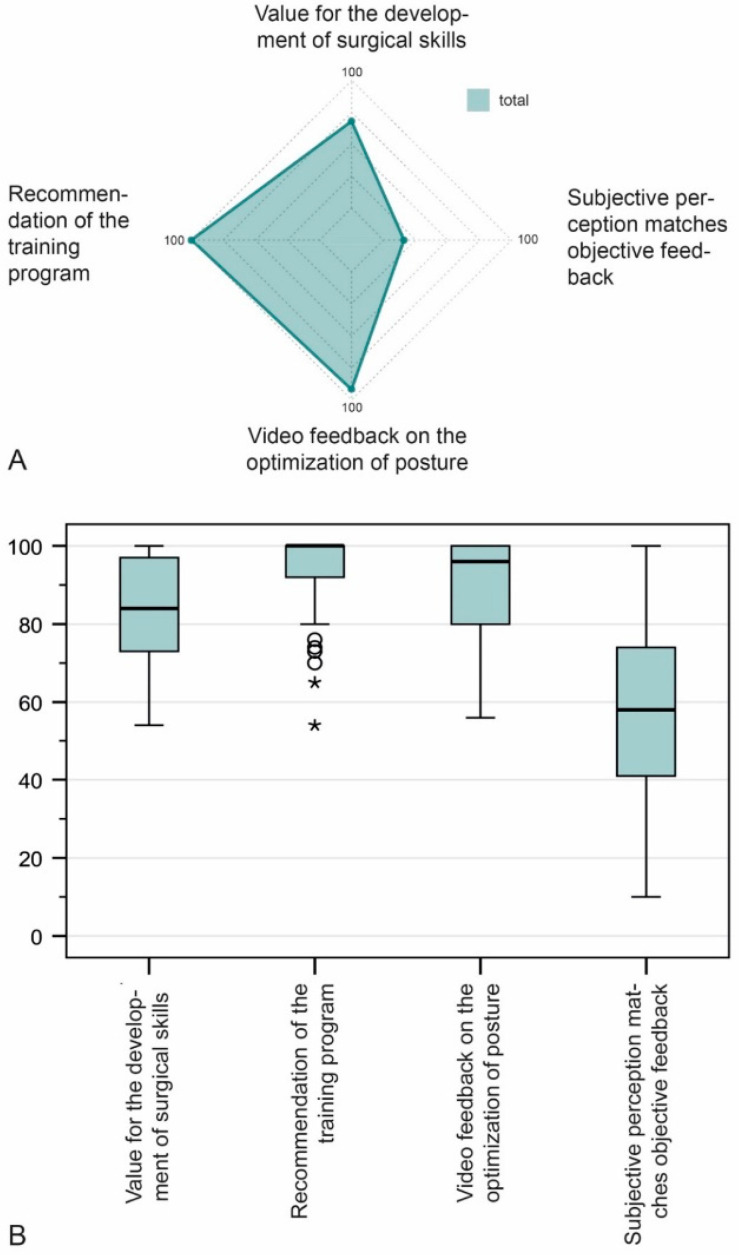
Evaluation of the course by all participants on the radar plot, with regard to four questions plotted on the x-axis. For better illustration, only the medians 50 to 100 are shown (**A**). The box plots show the evaluation of all participants. Circles and stars represent outliers; the black horizontal line within the box plot is the median (**B**).

The median agreement of the attendees regarding the value of the training was “very high” (80–100%) (Figure 5A). The median value was used because the attendees differed vastly in terms of age, surgical experience, and other variables. Further details are shown in Figure 5B. The median rating for the value for the surgical curriculum was 84% (IQR 72.5–97.5%, *n* = 37). The attendees said they would recommend a training course of this nature (median 100%, IQR 89.5–100%, *n* = 37). Similar ratings was given to the question as to whether the exercise improved their posture (median 96%, IQR 80–100%, *n* = 37). Concurrence of the attendees’ self-assessment with the expert’s assessment was expressed as follows: median 58%, IQR 40.5–76%, *n* = 37 (Figure 5B).

We also analyzed the attendees’ level of training, divided into residents and specialists.

**Figure 6:**
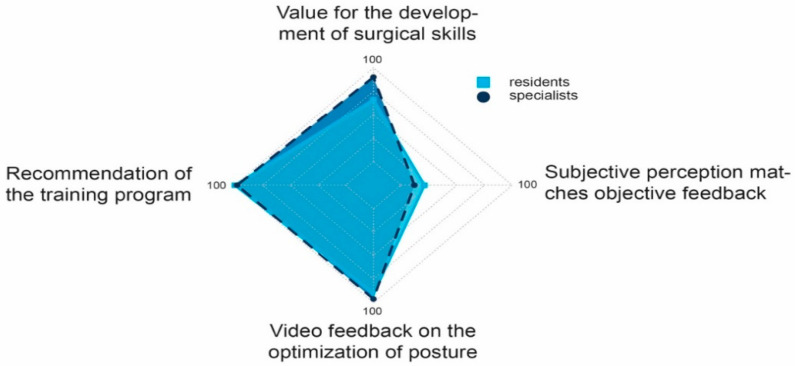
Median ratings of residents and specialists with regard to four questions plotted on the x-axis. For better illustration, only the medians 50 to 100 are shown.

**Figure 7:**
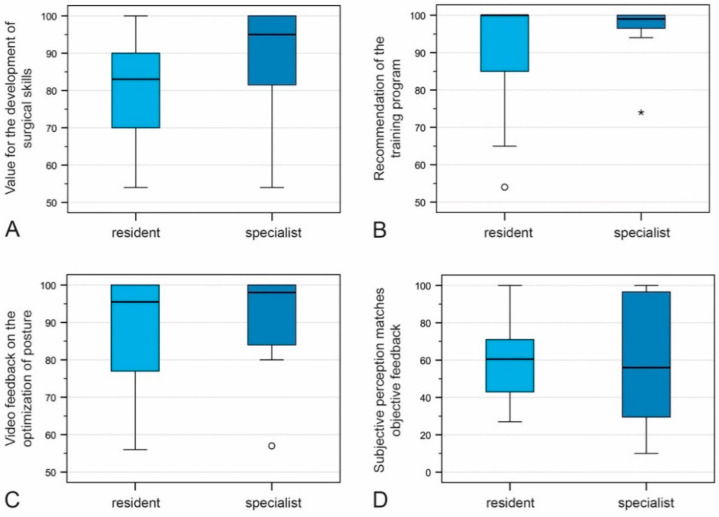
Residents’ and specialists’ assessment of the value of the training course for the development of surgical skills (**A**), whether they would recommend video feedback training (**B**), whether video feedback helps to optimize their posture (**C**), and whether the attendee’s subjective assessment concurs with the expert’s objective assessment (**D**). Circles and stars represent outliers; the black horizontal line within the box plot is the median.

The median agreement of the attendees with regard to the value of the training course for the development of surgical skills was 95% among residents (IQR 80–100%, *n* = 26) and 83% among specialists (IQR 69.75–90%, *n* = 11). Both, residents and specialists would recommend such training to others (median 99%, IQR 96–100%, *n* = 11; median 100%, IQR 83.75–100%, *n* = 11). Improvement of posture by video feedback was given a median rating of 98% (IQR 81–100%, *n* = 26) and 95.5% (IQR 76.75–100%, *n* = 11) by resident doctors and specialists, respectively. Concurrence of the attendees’ subjective feedback with the experts’ objective feedback was 56% (IQR 27–100%, *n* = 26) and 60.5% (IQR 42.50–71%, *n* = 11) for residents and specialists, respectively. None of the four variables differed significantly between the two groups (U test, *p* ≥ 0.05) (Figure 6 and Figure 7A–D).

**Figure 8:**
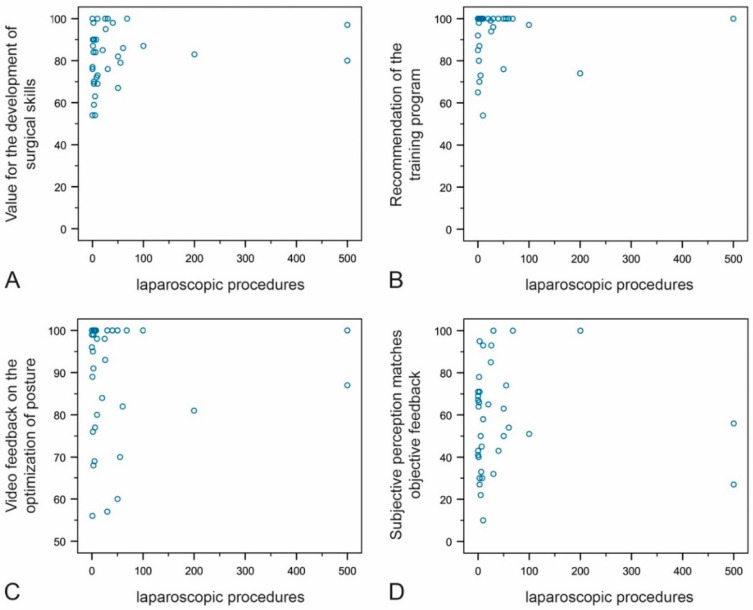
Correlation of the number of laparoscopic interventions and the value of the course for the development of surgical skills (**A**), the recommendation of video feedback training (**B**), the benefit of video feedback for posture (**C**), and whether the attendees’ subjective assessment is equivalent to the experts’ objective feedback (**D**). One attendee who had performed 2000 procedures was not listed.

We also performed a subgroup analysis of the number of laparoscopic interventions and the four variables. The value of video feedback for the development of surgical skills and the recommendation of such training were insignificantly correlated with the number of performed laparoscopic surgeries (Spearman’s correlation coefficients were 0.189 and 0.147, respectively). The number of performed laparoscopic surgeries was not correlated with the value of the course for the improvement of posture; Spearman’s rank correlation coefficient was −0.014). Concurrence of the attendees’ subjective feedback with the experts’ objective feedback was also insignificantly correlated with the number of laparoscopic surgeries (Spearman’s rank correlation coefficient 0.166) (Figure 8A–D).

Based on the answers to the open questions, the most frequently cited advantage of attending the video feedback course was that the attendees’ strengths and weaknesses were clarified and rendered objective. Comments on hand position and posture were greatly appreciated. One attendee was initially apprehensive of video feedback and less apprehensive afterwards.

Some of the negative comments concerning video feedback and video modeling were that the attendees felt they worked under pressure due to the impending assessment, and had a sensation of being watched. One attendee admitted to fear of others’ reactions and stated that all of the person’s mistakes were probably not corrected. A representative selection of comments is shown in Table 3.

## 4. Discussion

We evaluated an innovative teaching concept in minimally invasive surgery. The positive effect of video feedback and video modeling on surgical training was independent of the attendees’ sociodemographic characteristics or their level of experience. The attendees’ subjective feedback varied considerably from that of video feedback with expert advice. It enabled us to visualize individual steps of the procedure, register the trainees’ mistakes, and correct these. This, we believe, is an important and hitherto neglected step in endoscopic training.

We adapted the concept of video feedback used in sports. Psychomotor skills result from the relationship between physical motion and cognition. In any sport, coordinated physical movements are needed to achieve a desired goal. Such coordination arises from cognition. Like sports, minimally invasive surgery requires psychomotor skills for good surgical performance. Video feedback and modeling are aimed toward psychomotor skills such as posture, weight distribution, body position, handling, and the surgeon’s static and pivotal movements.

Video feedback has been used fruitfully in sports. Oñate et al. investigated various feedback concepts based on kinetic analysis for jumping and landing exercises in basketball. The exercises were performed by 51 recreational athletes to improve their performance and prevent injury to the anterior cruciate ligament. The use of self-assessment and video-taped feedback rated by an expert were most valuable for the improvement of landing skills [23]. Gymnastics is a complex sport which requires several body movements and postures. At the University of South Florida, scientists investigated the effect of combined video feedback and video modeling by an expert in four gymnastics students. After performing a specific exercise, the students watched the video of an expert doing the exercise and their own recording of the exercise with feedback—all of the students improved their skill as a result of the intervention [27]. Feedback on posture was given, albeit without any sports science expertise. The purpose was to train awareness of body posture. Such awareness enhances a person’s awareness of his/her actions at the operating table as well.

Video feedback has been used for surgical training and has yielded various results. Farquharson et al. clearly showed the improvement of surgical skills by video feedback among undergraduate students [29]. Forty-eight persons were divided into two groups: group 1 received video feedback and group 2 received verbal feedback. Both groups performed the suture and knot technique. The OSATS (Objective Structured Assessment of Technical Skills) score in group 1 was significantly higher after video feedback (the mean score for the first performance was 12.33, and the mean score for the second performance 14.02; *p* = 0.002); the difference was statistically significant compared to group 2 (*p* < 0.001). Backstein et al. performed a quantitative and qualitative evaluation of the benefit of repeated video feedback among 26 first-year surgical residents. The control group received only expert advice and the experimental group received video feedback with expert advice. The MOSAT score (mini objective structured assessment of technical skills) was used for the evaluation. A score of 31.46 in the experimental group versus 29.75 in controls revealed no significant benefit in the former group [31]. Nesbitt compared the views of undergraduate medical students on standard lecture feedback, unsupervised video feedback, and supervised video feedback after the students had tied a reef knot with the aid of an instrument. As in our study, the students strongly recommended individual video feedback (IVF) over standard lecture feedback (SLF) and unsupervised video feedback (UVF)—the difference was significant (IVF vs. SLF, *p* = 0.001). The students also considered group feedback useful [32], as did our course attendees. Analogous to Nesbitt’s study and ours, residents rated the assessment positively in Backstein’s qualitative evaluation of video feedback. Residents commented on the fine-tuning of a particular task, the benefit of being able to visualize their errors, and believed that visualization would help in further stages once the basic task had been learned. Two residents in this study reported inconsistencies in the expert’s feedback during review of the video tapes from one week to the next. This is indicative of a learning curve for experts when using videotape to instruct students [31].

We used video feedback with the demonstration of an expert´s video to compare the recording of the attendee’s performance with that of the experts. The concept of video modeling proved to be advantageous in sports and music. Caliendo et al. made music band and choral students listen to and analyze professional recordings of music, and give a critique of their own videotaped performance. The comparison of pre- and post-test results showed an increase in music achievement scores from 79.6% to 90.2% for band students, and from 74.5% to 87.7% for choral students. In our study, one attendee commented on the positive value of being shown the expert video: it helped to fill in gaps in her performance [27,33].

The assignment of tasks for feedback and evaluation should be aligned to the attendee’s level of education. Farooq et al. evaluated the benefit of video feedback in laparoscopic pig gallbladder dissections among 16 medical students and first-year residents of surgery, and found it no better than traditional verbal feedback [34]. The reason was possibly the fact that surgical novices were given a complex task rather than a task aligned to their level of surgical training. Although the time given for the video review was twenty minutes, the attendees were unable to comprehend their mistakes without expert advice.

We selected one simple and one complex exercise in order to accommodate residents and specialists.

Singh et al. found video feedback to be beneficial in laparoscopic training, and the authors used five laparoscopic trials with 30 min of video watching in between them [35]. This investigation clearly showed that the attendees must be given adequate time to view the video. Tasks must be performed repeatedly to achieve improvement. Feedback was given to the group for two hours—each participant was evaluated personally for seven to ten minutes. The time frame was considered sufficient by the participants.

Since a person’s perception of his/her performance will be different after having viewed the same on a video, the change of perception will indicate the magnitude of improvement in a task. In our study, the attendee’s perception of the task differed markedly after the attendee had watched the video recording along with expert advice. The difference in perception was nearly 40% in both groups. The median assessment of residents (60.5%) and specialists (56%) were similar after watching the video.

Kardash et al. evaluated 26 medical students who were taught laryngoscopy with video feedback. Video feedback changed the students’ perception of their performance [36]. Objective video feedback with expert advice had a profound impact on the trainee’s personal perception of his/her task and provided significant scope for improvement.

Time and money may be notable issues in any type of additional training. In Nesbitt et al.’s study, students answered open questions in a questionnaire and remarked that a medical school lacks time and financial resources for individualized video feedback [32]. Abbott et al., who used personal video feedback to enhance learning the skill of laparoscopic knot tying, also concluded that video feedback is time consuming and probably costly as well [7]. However, individualized video feedback is feasible in a small setup as endoscopic training courses. Our sequential training course is effective and could possibly reduce the overall duration of training. It also saves time and reduces workload for teachers. The additional cost of video recording, the equipment, and the technical staff might increase the cost of these courses. Nevertheless, the incorporation of this type of a course is justified in view of its added benefit in training surgery students [37].

One limitation of the present study is that it only included the candidate’s subjective evaluation of his or her performance after the feedback. Furthermore, giving feedback in groups may come with a certain psychological affect. To counteract this assessment only by zooming on handling or by blurring of faces or using specific identification number can be done. Controlled studies with objective evaluations will be needed to confirm the benefits registered in this pilot study. Secondly, the study lacked a control group. Thirdly, despite the fact that we observed no negative effects, the sustainability of the effects described here will have to be checked empirically. Dynamic developments in video and information technology, such as virtual training and artificial intelligence suggest that high-quality tools will be available in the future. These will meet the requirements of minimally invasive teaching and support the learning process.

## 5. Conclusions

Video feedback and video modeling have been shown to produce effective results in a variety of applications from communication training to sports. We conclude that it is a promising and sophisticated tool for surgical training as well. Video feedback and video modeling give teachers a lucid view of the tasks performed by trainees. Aspects of the task which may have been missed in ordinary verbal feedback are seen more clearly in video feedback. It helps the trainee to register his/her mistakes and gives the trainee a better perspective of the task.

The combined use of video feedback and video modeling is a promising tool to improve the execution of complex skills in laparoscopic surgery, perform precise body motions, and assume the appropriate position for a task. Another aspect worthy of investigation is the expert’s learning curve in giving video feedback.

## Figures and Tables

**Table 1:** List of equipment used.

	Equipment
Settings	Camera (Panasonic, Osaka, Japan) Panasonic LUMIX GH5 with a fixed focal length lens Panasonic Summilux 1:1.4/25 Panasonic LUMIX G81 with Panasonic G Vario 1:3.5-5.6/12-60
	Memory card 2× 64 GB SanDisk Extreme PRO 95 MB/s
	Tripod Manfrotto 055PROB
	Light (optional) LED light Yongnuo Digital YN600L with tripod (Yongnuo, Shenzhen, Guangdong province, People’s Republic of China)
	Laptop MacBook Pro 2015 (Apple Inc., Cupertino, California, USA)
	Staff Filmmaker and training supervisor
Endoscopic system	Karl Storz NDS wide-view HD 26-in monitor with LED backlight (16:10) IMAGE 1 HUBTM HD (so that the camera could display in HD) Camera (H3-z Image 1 HD Camera Head with the HOPKINS Straight Forward telescope 0°).
Instruments	Karl Storz Clickline (KARL STORZ GmbH and Co. KG, Tuttlingen, Baden-Württemberg, Germany) Koh needle holder KARL STORZ GmbH and Co. KG, Tuttlingen, Baden-Württemberg, Germany)
Pelvitrainer	Pelvitrainer 2.0 Realsimulator (Endodevelop, Saarbrücken, Saarland, Germany) SZABO-BERCI-SACKIER pelvitrainer (KARL STORZ GmbH and Co. KG, Tuttlingen, Baden-Württemberg, Germany)
Software used	Adobe Premiere Pro CC (Adobe, San Jose, California, USA)
Video feedback	iPad air A1475
	Software: Coach’s Eye (Tech Smith Corp, Okemos, Michigan, USA)
	Smart board: Smart UX60 (SMART Technologies inc., Calgary, Alberta, Canada)

**Table 2:** Sociodemographic data and laparoscopic experience.

	*n*	Mean	Standard Deviation	Minimum	Maximum	Percentile		
						25.	50. (Median)	75.
Age (years)	35	34.5	7.1	25	56	30.0	33.0	35.0
Professional experience (years)	36	6.89	8.1	0.0	40.0	3.0	3.8	10.0
Total number of laparoscopic procedures as surgeon	37	103.6	340.5	0	2000	2.5	10.0	50.0

**Table 3:** Comments given by participants to the open questions in the questionnaire.

What Did You Like?	What Did You Not Like?	What Should Be Done Differently?
“It improves self-awareness / self-assessment.” “Constructive criticism from experienced surgeons.” “Seeing the expert’s demonstration and listening to the explanation help to improve my skills.” “Seeing yourself from different perspectives improves handling and posture.” “Learning from others´ mistakes helps to improve oneself.” “There was enough time for the video feedback.”	“You are under pressure to complete the task as quickly as possible.” “Under pressure, one tries to perform the task quickly and pays less attention to quality.” “Not all mistakes were corrected.” “Fear of reactions from other participants.”	“Incorporation of breaks in the feedback.” “Re-evaluation of the performed task after video feedback.”

## Data Availability

The data presented in this study are available on request from the corresponding author. The data are not publicly available due to data privacy protection.

## Supplementary Materials

The following are available online at https://www.mdpi.com/2077-0383/10/1/163/s1, Video S1: Video feedback and video modeling clip.
